# Questionnaire survey of satisfaction with medication for five symptom domains of dementia with Lewy bodies among patients, their caregivers, and their attending physicians

**DOI:** 10.1111/psyg.12993

**Published:** 2023-06-25

**Authors:** Shunji Toya, Yuta Manabe, Mamoru Hashimoto, Hajime Yamakage, Manabu Ikeda

**Affiliations:** ^1^ Medical Science Sumitomo Pharma Co., Ltd. Tokyo Japan; ^2^ Department of Dementia and Geriatric Medicine, Division of Clinical Science Kanagawa Dental University School of Dentistry Yokosuka Japan; ^3^ Department of Psychiatry Osaka University Graduate School of Medicine Osaka Japan; ^4^ Department of Neuropsychiatry Kindai University Faculty of Medicine Osaka Japan; ^5^ Insight Clinical Development Group 3H Medi Solution Inc. Toshima‐ku Tokyo Japan

**Keywords:** caregivers, patients with DLB, pharmacotherapy, physicians, satisfaction, surveys and questionnaires

## Abstract

**Background:**

The real‐world status of satisfaction with medication for dementia with Lewy bodies (DLB) has not been elucidated. We assessed the satisfaction of patients with DLB, their caregivers, and their attending physicians (trios) with medication according to the clinical symptom domains of DLB.

**Methods:**

This was a subanalysis of a cross‐sectional, questionnaire‐based, survey study of trios. The subanalysis set comprised analysis populations for cognitive impairment, parkinsonism, psychiatric symptoms, sleep‐related disorders, and autonomic dysfunction (orthostatic hypotension, constipation, and dysuria). These analysis populations included trios of patients who had any symptom domain and took medication for each symptom domain, and for which all trio data on satisfaction with medication for the symptom domain were available. The degrees of satisfaction with medication were classified as ‘satisfied’, ‘neutral’, or ‘dissatisfied’.

**Results:**

The analysis set for this study included 110 trios for cognitive impairment, 62 for parkinsonism, 47 for psychiatric symptoms, 29 for sleep‐related disorders, none for orthostatic hypotension, 11 for constipation, and seven for dysuria. There were no statistically significant differences in the degree of satisfaction with medication for symptom domains other than parkinsonism and dysuria between patients–caregivers, patients–physicians, and caregivers–physicians. Regarding satisfaction with medication for parkinsonism, significantly more physicians than patients answered ‘satisfied’ (75.8% vs. 51.6%), and significantly more patients than physicians answered ‘neutral’ (35.5% vs. 14.5%) (*P* = 0.013). Regarding satisfaction with medication for dysuria, significantly more caregivers than physicians answered ‘satisfied’ (100% vs. 28.6%, *P* = 0.038).

**Conclusions:**

Satisfaction with medication for symptom domains other than parkinsonism and dysuria was similar among trios. Our results suggest that physicians should pay more attention to patients' satisfaction with medication for parkinsonism, and to caregivers' satisfaction with medication for dysuria to help prevent undermedication.

## INTRODUCTION

Dementia with Lewy bodies (DLB) is the second most common neurodegenerative dementia in patients after Alzheimer's disease (AD).^1^, ^2^, ^3^

DLB is an intractable disease for which there is no fundamental cure, although there are several options for symptom‐relieving medication. Patients with DLB have a wide variety of symptoms.^4^ While attending physicians use pharmacological and non‐pharmacological therapies to treat the range of clinical symptoms of DLB, pharmacological interventions for specific symptoms are limited for various reasons such as hypersensitivity to antipsychotic medications and the lack of medication with high efficacy and safety for target symptoms.^4^, ^5^, ^6^ For example, psychotic symptoms may worsen with the use of some antiparkinsonian drugs,^7^ and parkinsonism may worsen with the use of antipsychotic drugs.^8^

Among various diseases, DLB is one of the most representative of unmet medical needs because the only drugs approved for treating DLB in Japan are donepezil for inhibiting cognitive deterioration and zonisamide as an adjunct to levodopa for parkinsonism. While there is a clear disease burden for patients with DLB and their caregivers,^9^ the real‐world status of satisfaction with medication for DLB is not known. Additionally, the attending physicians' degree of satisfaction with the medication that they prescribe for symptoms of DLB is also unknown.

We previously conducted a multicentre, cross‐sectional, observational survey study of the treatment needs of DLB according to patients, their caregivers, and their attending physicians, and we identified the treatment needs of patients and caregivers and the perceptions of their physicians regarding these medical needs.^10^ This subanalysis assessed the satisfaction of the study participants with medication for five symptom domains of DLB: cognitive impairment, parkinsonism, psychiatric symptoms, sleep‐related disorders, and autonomic dysfunction (orthostatic hypotension, constipation, and dysuria).

## METHODS

### Study design

The main study was conducted from September 2020 to July 2021 and the details of the study design have been previously reported.^10^ This study was conducted at 35 facilities in Japan including university hospitals, non‐university hospitals, and clinics.

This study was conducted in accordance with the ethical principles based on the Declaration of Helsinki (revised in 2013), the Ethical Guidelines for Medical and Health Research Involving Human Subjects (partially revised in 2017), and the research protocol. This study was approved by the Ethical Review Committee for Observational Research of Osaka University Hospital and the respective Ethical Review Committees of the other study sites. This study was registered at UMIN Clinical Trials Registry (UMIN000041844). Written informed consent was obtained from all the study participants (patients, caregivers, and attending physicians).

### Participants

A set of three participants (patient–caregiver–physician) was defined as a trio. The inclusion criteria for patients with DLB were as follows: outpatients with a diagnosis of probable DLB, aged 50 years or older, and those whose attending physician had been treating them for at least 3 months. Patients whose attending physicians deemed them unsuitable for the study were excluded. The inclusion criteria for caregivers were as follows: primary caregivers who were primarily responsible for caring for patients with DLB and aged 20 years or older. The inclusion criteria for the attending physicians were that they had to be experts in DLB treatment in Japan, defined as previously reported.^10^

This subanalysis set comprised analysis populations for cognitive impairment, parkinsonism, psychiatric symptoms, sleep‐related disorders, and autonomic dysfunction (orthostatic hypotension, constipation, and dysuria). These analysis populations contained trios in which patients had at least one symptom domain and took medication for each symptom domain, and for which all trio data on satisfaction with medication for that symptom domain were available.

### Contents of the questionnaire for this research

Questionnaires were individually prepared for patients, caregivers, and physicians, the details of which have been reported.^10^ In this study, 52 symptoms that are frequent and clinically important in DLB were pre‐selected and further classified into seven symptom domains.^10^ The questionnaire choices for patients and caregivers included seven items: ‘very satisfied’, ‘satisfied’, ‘neither’, ‘unsatisfied’, ‘very unsatisfied’, ‘no medication’, and ‘unknown’ to evaluate their satisfaction with the effectiveness of their current medication for the five symptom domains of cognitive impairment, parkinsonism, psychiatric symptoms, sleep‐related disorders, and autonomic dysfunction (orthostatic hypotension, constipation, and dysuria). The questionnaire choices for physicians included six items: ‘very satisfied’, ‘satisfied’, ‘neither’, ‘unsatisfied’, ‘very unsatisfied’, and ‘no medication’, to evaluate their satisfaction with the effectiveness of the current medication for the five symptom domains. The types of medication prescribed to patients with the five symptom domains were assessed from the questionnaire for physicians (Q26 to Q30 for attending physician).

Medications for cognitive impairment, parkinsonism, psychiatric symptoms, and sleep‐related disorders included all those that physicians reported in response to the question in the physician questionnaire on prescriptions for the treatment of each symptom domain. Medications for autonomic dysfunction included the following: for orthostatic hypotension, droxidopa, midodrine, amezinium, and propranolol; for constipation, intestinal secretagogues, elobixibat, osmotic laxative, bulk‐forming laxative, irritant laxative, gastrointestinal prokinetic agent, Chinese herbal medicine except irritant laxatives, suppository/enema, and other agents for digestive issues; and for dysuria, therapeutic agents for irritative symptoms (e.g. frequency, urgency, and urge incontinence) and obstructive voiding symptoms (e.g. hesitancy and weak urinary stream).

### Assessments and patients' symptoms

Patients and caregivers were screened using the Japanese version of the Mini‐Mental State Examination (MMSE‐J),^11^ the Japanese version of the Movement Disorder Society‐Unified Parkinson's Disease Rating Scale (MDS‐UPDRS) Part III,^12^ the Japanese version of the Neuropsychiatric Inventory‐12 (NPI‐12),^13^ the Cognitive Fluctuation Inventory,^14^ and a shortened Japanese version of the Zarit Caregiver Burden Interview.^15^ They were then asked to watch a video explaining the clinical symptoms of DLB. Finally, the patients and caregivers each received questionnaires that they were to complete and submit within 3 weeks. To avoid inaccuracies in the patients' answers due to dementia or parkinsonism (e.g. tremor), the caregivers were allowed to assist the patients in completing the questionnaire, after completing their own questionnaires first. Physicians answered the survey for each patient, which included questions on their degree of satisfaction with medications, via the internet.

Patients' symptom domains were defined as follows: cognitive impairment was defined as memory impairment, disorientation, executive dysfunction, attention dysfunction, fluctuating recognition, visuospatial dysfunction, or other symptoms of cognitive impairment identified by the physician in the physician questionnaire (Q39); parkinsonism was defined as having an MDS‐UPDRS Part III total score ≥7 (including all sub‐items:^12^, ^16^ rest tremor of the hands (3.15), kinetic tremor of the hands (3.16), facial expression (3.2), global spontaneity of movement (body bradykinesia) (3.14), and rigidity (3.3)) or having one MDS‐UPDRS Part III sub‐item score ≥3;^17^ psychiatric symptoms were defined as having an NPI‐10 score ≥1,^18^ excluding night‐time behaviour and appetite and eating abnormalities from NPI‐12; sleep‐related disorder was defined as having an NPI‐12 night‐time behaviour sub‐item score ≥1; and autonomic dysfunction (which included orthostatic hypotension, constipation, and dysuria) was defined as having symptoms of orthostatic hypotension, constipation, or dysuria as reported by the physician in the physician questionnaire (Q39).

### Statistical analysis

Summary statistics were calculated as mean ± standard deviation (SD) for continuous scales, and frequency and percentage for nominal scales. To compare satisfaction with medication for the five symptom domains, including the three symptoms of autonomic dysfunction among the participant trios, the answers were reclassified as follows: ‘very satisfied’ and ‘satisfied’ to ‘satisfied’, ‘neither’ to ‘neutral’, and ‘unsatisfied’ and ‘very unsatisfied’ to ‘dissatisfied’.

Participants who did not answer the question regarding their satisfaction with medication were excluded. If patients and caregivers answered ‘no medication’ or ‘unknown’, and physicians answered ‘no medication’, the corresponding trio was also excluded.

The Wilcoxon signed‐rank test was used to compare the frequency distribution of ‘satisfied’, ‘neutral’, and ‘dissatisfied’ in participant trios. Statistical significance was set at 0.05 on both sides, and all analyses were performed with SAS ver. 9.4 (Statistical Analysis Software; SAS Institute Inc., Cary, NC, USA).

## RESULTS

### Participants

The background characteristics of patients with DLB, their caregivers, and their attending physicians (full analysis set) have been previously reported.^10^ Briefly, the mean ± SD age of patients with DLB was 79.3 ± 6.7 years, and the duration after diagnosis of DLB was 30.4 ± 29.7 months. The mean ± SD MMSE‐J, NPI‐12, and MDS‐UPDRS Part III total scores were 20.9 ± 5.9, 16.0 ± 16.6, and 23.8 ± 20.6, respectively. The caregivers had a mean ± SD age of 64.8 ± 12.8 years, 191 (72.6%) were female, 139 (52.9%) were spouses of the patients, and 104 (39.5%) were children of the patients. The attending physicians had a mean ± SD age of 51.1 ± 7.8 years. Psychiatry was the most common affiliated clinical department (69.4%, *n* = 26), and >50% (*n* = 21) of the physicians had treated >100 patients with DLB.

**Figure 1:**
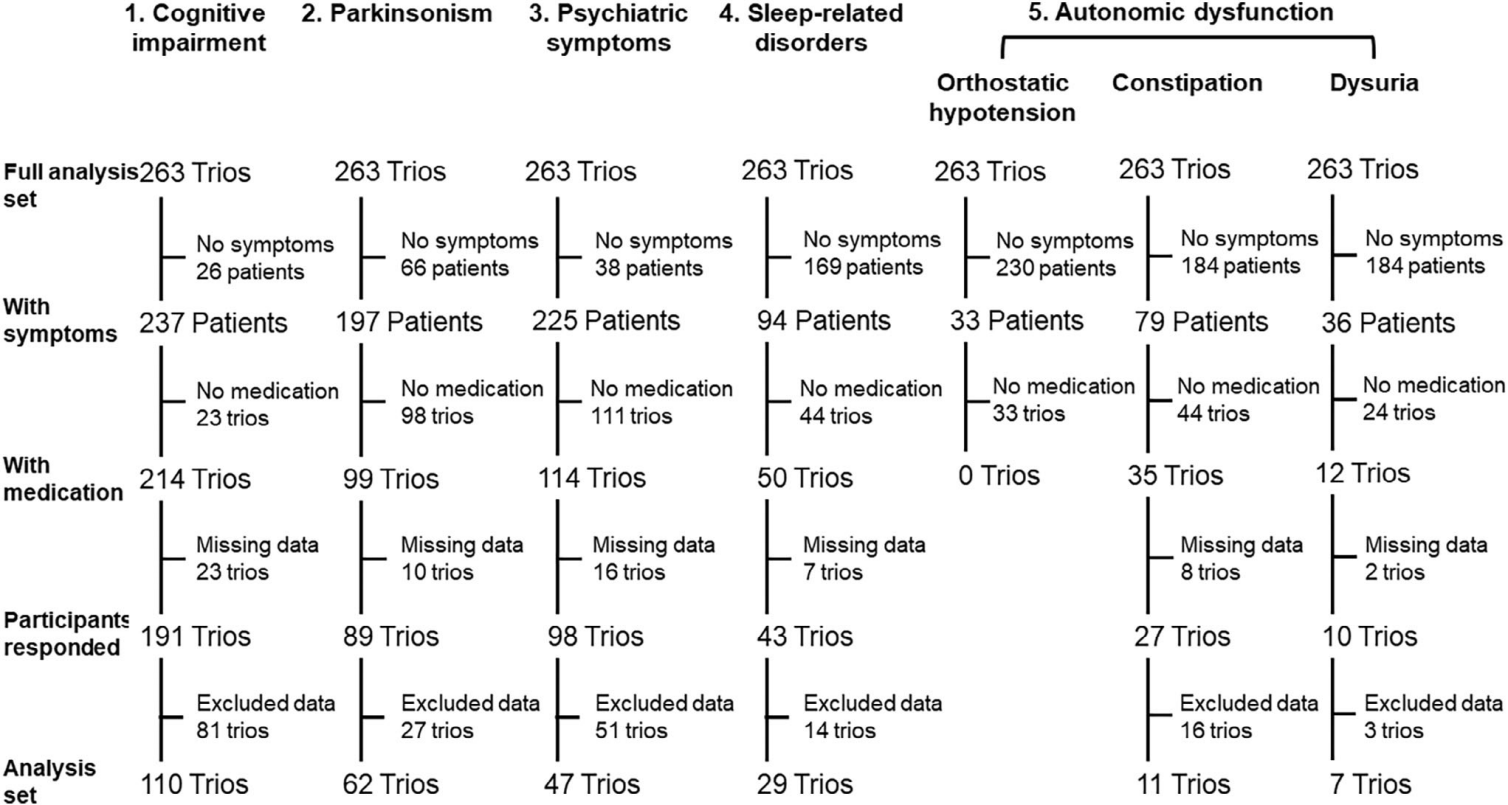
Disposition of the study participants. The full analysis set consisted of 263 patient–caregiver–physician trios. ‘No symptoms’ means that the patient did not have symptoms corresponding to one of the five symptom domains in the column headings. Note that one patient had multiple symptoms (both constipation and dysuria).

The disposition of the study participants is shown in Fig. 1. Of the 263 patients in the full analysis set of the main study, the numbers of patients with cognitive impairment, parkinsonism, psychiatric symptoms, sleep‐related disorders, orthostatic hypotension, constipation, and dysuria were 237, 197, 225, 94, 33, 79, and 36, respectively. Of these, the numbers of trios in which patients were receiving pre‐defined medication for each of the above symptom domains were 214, 99, 114, 50, 0, 35, and 12, respectively. The study excluded participants with no available data on satisfaction with medication and included only trios in which all three participants had available data on satisfaction with medication in the analysis set for this subanalysis. Finally, the analysis set included 110 trios (33 physicians) for cognitive impairment, 62 trios (23 physicians) for parkinsonism, 47 trios (19 physicians) for psychiatric symptoms, 29 trios (19 physicians) for sleep‐related disorders, zero trios for orthostatic hypotension, 11 trios (four physicians) for constipation, and seven trios (four physicians) for dysuria. For orthostatic hypotension, no patient was prescribed a pre‐defined medication in this study, thus, the satisfaction with medication for orthostatic hypotension could not be evaluated in this study.

### Satisfaction with the medication for cognitive impairment

The background characteristics of the 110 patients with cognitive impairment and their caregivers for cognitive impairment are shown in Table 1. The mean ± SD MMSE‐J total score was 21.3 ± 5.0. Cholinesterase inhibitors were the most frequently prescribed medication (108 (98.2%) patients) including donepezil (80 (72.7%)), galantamine (9 (8.2%)), and rivastigmine (19 (17.3%)). Other prescribed medications for cognitive impairment were memantine (14 (12.7%)) and cilostazol (one (0.9%)).

**Table 1:** Background characteristics of patients with each symptom domain or symptom and their caregivers

	Cognitive impairment *n* = 110	Parkinsonism *n* = 62	Psychiatric symptoms *n* = 47	Sleep‐related disorders *n* = 29	Constipation *n* = 11	Dysuria *n* = 7
Patients' age, years	80.2 ± 6.4	78.4 ± 6.7	79.1 ± 6.7	79.1 ± 7.1	79.9 ± 6.3	81.3 ± 8.1
Patients' gender, male/female	49/61	32/30	17/30	13/16	7/4	6/1
Duration after diagnosis of DLB, months	29.1 ± 25.8	38.0 ± 34.6	36.4 ± 38.8	34.7 ± 32.3	53.1 ± 61.6	64.4 ± 47.5
Patient education, years	11.9 ± 3.0	11.8 ± 3.3	11.6 ± 2.8	11.9 ± 3.2	12.0 ± 3.7	12.4 ± 5.0
MMSE‐J total score	21.3 ± 5.0	21.7 ± 5.4	20.8 ± 5.3	21.4 ± 4.4	21.9 ± 4.8	20.0 ± 6.5
NPI‐12 total score	17.6 ± 16.4	14.5 ± 14.9	23.0 ± 18.6	26.6 ± 14.6	15.5 ± 13.9	23.0 ± 12.4
NPI‐10 total score	—	—	17.4 ± 14.9	—	—	—
Night‐time behaviour subdomain	—	—	—	5.1 ± 2.9	—	—
MDS‐UPDRS Part III total score	21.2 ± 17.6	37.7 ± 19.9	23.9 ± 16.5	29.9 ± 26.6	33.4 ± 27.0	33.1 ± 17.2
CFI score	2.5 ± 3.1	2.2 ± 3.4	2.9 ± 3.4	3.5 ± 3.9	1.8 ± 2.4	3.7 ± 4.6
J‐ZBI‐8 score	9.1 ± 6.4	9.2 ± 6.7	10.6 ± 6.6	12.6 ± 7.6	10.4 ± 6.3	13.3 ± 8.0
Caregivers' age, years	65.0 ± 11.5	63.5 ± 12.2	63.7 ± 12.8	61.8 ± 12.7	59.3 ± 13.5	59.4 ± 14.0
Caregivers' gender, male/female	31/79	13/49	19/28	6/23	2/9	0/7

*Note*: Data are *n* (%), mean ± standard deviation, or *n*/*n*.

Abbreviations: CFI, Cognitive Fluctuation Inventory; DLB, dementia with Lewy bodies; J‐ZBI‐8, Japanese version of the Zarit Caregiver Burden Interview; MDS‐UPDRS, Movement Disorder Society‐Unified Parkinson's Disease Rating Scale; MMSE‐J, Japanese version of the Mini‐Mental State Examination; NPI‐12, Neuropsychiatric Inventory‐12; NPI‐10, Neuropsychiatric Inventory‐10.

Satisfaction with medication for cognitive impairment according to the study participants is shown in Table 2. The numbers of patients, caregivers, and physicians who answered being ‘satisfied’ with medication for cognitive impairment were 69 (62.7%), 76 (69.1%), and 80 (72.7%), respectively. There were no statistically significant differences in the degree of satisfaction between patients–caregivers, patients–physicians, and caregivers–physicians (*P* = 0.062, 0.466, and 0.497, respectively).

**Table 2:** Satisfaction with medication for each symptom domain of dementia with Lewy bodies according to the patient, their caregiver, and their attending physician

OC	AR	Patient		Caregiver		Physician		*P*‐values
Cognitive impairment (*n* = 110 trios)								
Very satisfied	Satisfied	21 (19.1)	69 (62.7)	26 (23.6)	76 (69.1)	22 (20.0)	80 (72.7)	Patient vs. Caregiver, *P* = 0.062
Satisfied		48 (43.6)		50 (45.5)		58 (52.7)		
Neither	Neutral	35 (31.8)	35 (31.8)	33 (30.0)	33 (30.0)	15 (13.6)	15 (13.6)	Patient vs. Physician, *P* = 0.466
Unsatisfied	Dissatisfied	6 (5.5)	6 (5.5)	1 (0.9)	1 (0.9)	6 (5.5)	15 (13.6)	Caregiver vs. Physician, *P* = 0.497
Very unsatisfied		0 (0.0)		0 (0.0)		9 (8.2)		
Parkinsonism (*n* = 62 trios)								
Very satisfied	Satisfied	5 (8.1)	32 (51.6)	10 (16.1)	34 (54.8)	8 (12.9)	47 (75.8)	Patient vs. Caregiver, *P* = 0.283
Satisfied		27 (43.5)		24 (38.7)		39 (62.9)		
Neither	Neutral	22 (35.5)	22 (35.5)	25 (40.3)	25 (40.3)	9 (14.5)	9 (14.5)	Patient vs. Physician, *P* = 0.013
Unsatisfied	Dissatisfied	5 (8.1)	8 (12.9)	3 (4.8)	3 (4.8)	5 (8.1)	6 (9.7)	Caregiver vs. Physician, *P* = 0.407
Very unsatisfied		3 (4.8)		0 (0.0)		1 (1.6)		
Psychiatric symptoms (*n* = 47 trios)								
Very satisfied	Satisfied	9 (19.1)	31 (66.0)	13 (27.7)	31 (66.0)	9 (19.1)	35 (74.5)	Patient vs. Caregiver, *P* = 0.967
Satisfied		22 (46.8)		18 (38.3)		26 (55.3)		
Neither	Neutral	14 (29.8)	14 (29.8)	13 (27.7)	13 (27.7)	9 (19.1)	9 (19.1)	Patient vs. Physician, *P* = 0.948
Unsatisfied	Dissatisfied	1 (2.1)	2 (4.3)	3 (6.4)	3 (6.4)	2 (4.2)	3 (6.4)	Caregiver vs. Physician, *P* = 0.527
Very unsatisfied		1 (2.1)		0 (0.0)		1 (2.1)		
Sleep‐related disorders (*n* = 29 trios)								
Very satisfied	Satisfied	6 (20.7)	19 (65.5)	6 (20.7)	21 (72.4)	5 (17.2)	19 (65.5)	Patient vs. Caregiver, *P* = 0.062
Satisfied		13 (44.8)		15 (51.7)		14 (48.3)		
Neither	Neutral	9 (31.0)	9 (31.0)	6 (20.7)	6 (20.7)	8 (27.6)	8 (27.6)	Patient vs. Physician, *P* = 0.983
Unsatisfied	Dissatisfied	1 (3.4)	1 (3.4)	1 (3.4)	2 (6.9)	2 (6.9)	2 (6.9)	Caregiver vs. Physician, *P* = 0.174
Very unsatisfied		0 (0.0)		1 (3.4)		0		
Constipation (*n* = 11 trios)								
Very satisfied	Satisfied	0 (0.0)	6 (54.5)	1 (9.1)	9 (81.8)	1 (9.1)	6 (54.5)	Patient vs. Caregiver, *P* = 0.102
Satisfied		6 (54.5)		8 (72.7)		5 (45.5)		
Neither	Neutral	2 (18.2)	2 (18.2)	1 (9.1)	1 (9.1)	4 (36.4)	4 (36.4)	Patient vs. Physician, *P* = 0.862
Unsatisfied	Dissatisfied	3 (27.3)	3 (27.3)	1 (9.1)	1 (9.1)	1 (9.1)	1 (9.1)	Caregiver vs. Physician, *P* = 0.999
Very unsatisfied		0 (0.0)		0 (0.0)		0 (0.0)		
Dysuria (*n* = 7 trios)								
Very satisfied	Satisfied	0 (0.0)	3 (42.9)	1 (14.3)	7 (100.0)	1 (14.3)	2 (28.6)	Patient vs. Caregiver, *P* = 0.059
Satisfied		3 (42.9)		6 (85.7)		1 (14.3)		
Neither	Neutral	3 (42.9)	3 (42.9)	0 (0.0)	0 (0.0)	2 (28.6)	2 (28.6)	Patient vs. Physician, *P* = 0.408
Unsatisfied	Dissatisfied	1 (14.3)	1 (14.3)	0 (0.0)	0 (0.0)	3 (42.9)	3 (42.9)	Caregiver vs. Physician, *P* = 0.038
Very unsatisfied		0 (0.0)		0 (0.0)		0 (0.0)		

*Note*: Data are *n* (%). *P*‐values for differences in degree of satisfaction between patients–caregivers, patients–physicians, and caregivers–physicians were calculated using the Wilcoxon signed‐rank test.

Abbreviations: AR, after reclassification; OC, original classification.

### Satisfaction with the medication for parkinsonism

The background characteristics of the 62 patients with parkinsonism and their caregivers are shown in Table 1. The mean ± SD MDS‐UPDRS Part III total score was 37.7 ± 19.9. The most frequently prescribed medication for parkinsonism was levodopa (61 (98.4%) patients), followed by zonisamide (13 (21.0%)), dopamine agonists (12 (19.4%)), monoamine oxidase B inhibitors (eight (12.9%)), catechol‐O‐methyltransferase inhibitors (five (8.1%)), amantadine (three (4.8%)), istradefylline (three (4.8%)), anticholinergic agents (one (1.6%)), and droxidopa (one (1.6%)).

Satisfaction with medication for parkinsonism according to the study participants is shown in Table 2. The numbers of patients, caregivers, and physicians who answered being ‘satisfied’ with medication for parkinsonism were 32 (51.6%), 34 (54.8%), and 47 (75.8%), respectively. There were no statistically significant differences in the degree of satisfaction between patients–caregivers and caregivers–physicians (*P* = 0.283 and 0.407, respectively). However, significantly more physicians than patients answered ‘satisfied’ (75.8% vs. 51.6%), and significantly more patients than physicians answered ‘neutral’ (35.5% vs. 14.5%) (*P* = 0.013).

### Satisfaction with the medication for psychiatric symptoms

The background characteristics of the 47 patients with psychiatric symptoms and their caregivers are shown in Table 1. The ratio of females (*n* = 30) to males (*n* = 17) was higher in this group than in the other groups. The mean ± SD NPI‐10 score was 17.4 ± 14.9. The most frequently prescribed medications for psychiatric symptoms were antidepressants and Yokukansan, a traditional Japanese herbal medicine, (both 15 (31.9%)), followed by antipsychotics (14 (29.8%)), cholinesterase inhibitors (eight (17.0%)), valproic acid (seven (3.1%)), carbamazepine (one (2.1%)), Chinese herbal medicine other than Yokukansan (one (2.1%)), and memantine (one (2.1%)). The antidepressant breakdown was selective serotonin reuptake inhibitors (11 (23.4%)), noradrenaline and specific serotonergic antidepressants (three (6.4%)), serotonin‐noradrenaline reuptake inhibitors (three (6.4%)), and tricyclic antidepressants (one (2.1%)).

Satisfaction with medication for psychiatric symptoms according to the study participants is shown in Table 2. The numbers of patients, caregivers, and physicians who answered being ‘satisfied’ with medication for psychiatric symptoms were 31 (66.0%), 31 (66.0%), and 35 (74.5%), respectively. There were no statistically significant differences in the degree of satisfaction between patients–caregivers, patients–physicians, and caregivers–physicians (*P* = 0.967, 0.948, and 0.527, respectively).

### Satisfaction with the medication for sleep‐related disorders

The background characteristics of the 29 patients with sleep‐related disorders and their caregivers are shown in Table 1. The mean ± SD NPI‐12 total score was 26.6 ± 14.6. The mean ± SD night‐time behaviour score of the NPI‐12 was 5.1 ± 2.9. The most frequently prescribed medications for sleep‐related disorders were benzodiazepines (11 (37.9%); including clonazepam, five (17.2%)), followed by orexin receptor antagonists (nine (31.0%); including lemborexant, six (20.7%), and suvorexant, three (10.3%)), non‐benzodiazepines (six (20.7%)), ramelteon (five (17.2%)), antidepressants (three (10.3%)), quetiapine (two (6.9%)), and Yokukansan (one (3.4%)).

Satisfaction with medication for sleep‐related disorders according to the study participants is shown in Table 2. The numbers of patients, caregivers, and physicians who answered being ‘satisfied’ with medication for sleep‐related disorders were 19 (65.5%), 21 (72.4%), and 19 (65.5%), respectively. There were no statistically significant differences in the degree of satisfaction between patients–caregivers, patients–physicians, and caregivers–physicians (*P* = 0.062, 0.983, and 0.174, respectively).

### Satisfaction with the medication for constipation

The background characteristics of the 11 patients with constipation and their caregivers are shown in Table 1. The ratio of males (*n* = 7) to females (*n* = 4) was higher in this group than in the other groups. Among the patients with constipation, one patient had symptoms of both constipation and dysuria. The most frequently prescribed medication for constipation was osmotic laxatives (10 (90.9%)), of which magnesium preparation was prescribed for nine patients (81.8%). Other frequently prescribed medications for constipation were irritant laxatives (three (27.3%)), followed by Chinese herbal medicines (Keishi‐ka‐shakuyakuto), elobixibat, intestinal secretagogues, polyethylene glycol, and other agents for digestive issues (each one (9.1%)).

Satisfaction with medication for constipation according to the study participants is shown in Table 2. The numbers of patients, caregivers, and physicians who answered being ‘satisfied’ with medication for constipation were six (54.5%), nine (81.8%), and six (54.5%), respectively. There were no statistically significant differences in the degree of satisfaction between patients–caregivers, patients–physicians, and caregivers–physicians (*P* = 0.102, 0.862, and 0.999, respectively).

### Satisfaction with the medication for dysuria

The background characteristics of the seven patients with dysuria and their caregivers are shown in Table 1. The mean ± SD NPI‐12 total score was 23.0 ± 12.4, which was numerically higher than that of patients with cognitive impairment (17.6 ± 16.4), parkinsonism (14.5 ± 14.9), and constipation (15.5 ± 13.9), but lower than that of patients with sleep‐related disorders (26.6 ± 14.6). All seven patients with dysuria received β3‐receptor agonists, of which mirabegron was prescribed in four (57.1%) patients. Other prescribed medications for dysuria were vibegron (three (42.9%)) and anticholinergic agent (imidafenacin) (one (14.3%)).

Satisfaction with medication for dysuria according to the study participants is shown in Table 2. The numbers of patients, caregivers, and physicians who answered being ‘satisfied’ with medication for dysuria were three (42.9%), seven (100.0%), and two (28.6%), respectively. There were no statistically significant differences in the degree of satisfaction between patients–caregivers and patients–physicians (*P* = 0.059 and 0.408, respectively). However, significantly more caregivers than physicians answered ‘satisfied’ (100% vs. 28.6%, *P* = 0.038).

## DISCUSSION

To the best of our knowledge, this is the first study to compare satisfaction with medication for cognitive impairment, parkinsonism, psychiatric symptoms, sleep‐related disorders, and autonomic dysfunction (constipation and dysuria) among patients with DLB, their caregivers, and their physicians. The present findings showed that there was no difference in satisfaction with medication for symptom domains other than parkinsonism and dysuria among the three types of participants. This suggests that the medicines currently on the market may provide adequate treatment for each DLB symptom and a reasonable degree of satisfaction among patients, their caregivers, and their physicians, although there are few approved drugs for treatment of DLB. However, the two main additional findings were that physicians were more likely than patients to be satisfied with medication for parkinsonism, and that caregivers were more likely than physicians to be satisfied with medication for dysuria.

Parkinsonism is a highly problematic symptom for patients and includes slowed movements (bradykinesia), impaired postural retention, gait disturbances, and tremor in the extremities, which affect activities of daily living.^19^, ^20^, ^21^, ^22^ Although there are clinical and neuropathological differences between DLB and Parkinson's disease (PD),^23^ previous reports indicate that there  is a significant gap between patients with PD and their physicians in satisfaction with treatment.^24^ A previous phase 3 study of rasagiline^25^ included patients with PD who presented with wearing‐OFF and used stable levodopa; the baseline mean ± SD MDS‐UPDRS Part III scores were 28.7 ± 13.28 and 27.5 ± 13.09 in the ON state in the rasagiline 0.5 mg/day and 1 mg/day groups, respectively. Patients presumably participated in that clinical trial expecting improvement in wearing‐OFF and activities of daily living. In the present study, patients who reported satisfaction with medication for parkinsonism showed higher MDS‐UPDRS Part III scores than those in the rasagiline clinical trial (37.7 ± 19.9).^25^ Patients in the present subanalysis may have anticipated further improvement in their activities of daily living after being prescribed medication for parkinsonism. A previous study of patients with PD reported no correlation between subjective ratings of improvement in parkinsonism and the degree of objective change in parkinsonian impairment or disability in patients with PD.^26^ These findings suggest that attending physicians should interview their patients about their satisfaction with medication for parkinsonism as well as the severity of their parkinsonism symptoms, and pay more attention to ensuring that patients with parkinsonism are not undermedicated.

In the present subanalysis, satisfaction with medication for dysuria was significantly higher among caregivers than attending physicians (*P* = 0.038). The high degree of caregiver satisfaction with medication for dysuria may have been because of the decrease in the frequency of urination and subsequent decrease in caregiver burden after medication for dysuria was administered to the patients. Although the satisfaction with medication was not significantly different between patients and caregivers, patient satisfaction with medication for dysuria appeared to be low. Overactive bladder is a common problem in patients with DLB,^27^ and one study suggests that symptoms of urgency and urge incontinence, associated with detrusor overactivity, are more prevalent in DLB than in other cognitive disorders such as PD with dementia and AD.^28^ Therefore, it is suggested that physicians should be more careful when communicating with caregivers regarding the effectiveness of, and their satisfaction with, medication for dysuria‐related disorders. However, this finding should be interpreted with caution because of the small sample size.

Unlike the findings on satisfaction with medication for parkinsonism and dysuria, in this subanalysis, satisfaction with medication for cognitive impairment, psychiatric symptoms, sleep‐related disorders, and constipation did not differ between patients, caregivers, and physicians. In a previous report that investigated medical needs by disease,^29^ satisfaction with medication was approximately 40% for patients with PD and approximately 25% for patients with AD, although satisfaction for patients with DLB was not reported. Satisfaction with medication for parkinsonism in the present study (51.6%) was broadly consistent with that for PD in the previous report, and that for cognitive impairment (62.7%) in the present study was likely higher than that for AD in the previous report. Most (98.2%) patients with DLB who had cognitive impairment were taking cholinesterase inhibitors, which may have contributed to the high treatment satisfaction in the present study. However, it should be noted that some patients and caregivers answered ‘neutral’ or ‘dissatisfied’ when asked about their satisfaction with medication for these symptoms.

Lack of awareness of visual hallucinations^30^ and memory impairment^31^ have been reported in patients with DLB. The pharmacological treatment of DLB can be extremely challenging for attending physicians, particularly when there is a need to manage parkinsonism in combination with hallucinations and delusions, and cognitive impairment in combination with dysuria. Based on the results of this subanalysis, attending physicians should consider whether treatment for these symptoms is needed even if the patients do not express having concerns with these symptoms because of a lack of awareness, and work in conjunction with patients and caregivers to increase their degree of satisfaction with medication for DLB. The development of novel therapeutic agents for DLB is still needed, and we hope the results of this study will provide some incentive for the development of novel therapeutic agents for DLB in the future.

The findings of the main study indicated that attending physicians should pay more attention to autonomic dysfunction and sleep‐related disorders in the treatment of DLB based on patients' treatment needs and physician perceptions.^10^ The findings also show that attending physicians should pay more attention to satisfaction with medication for parkinsonism. Furthermore, satisfaction with medication for dysuria was higher among caregivers and lower among physicians in the present study. This result was based on the evaluations by physicians who prescribed medication for autonomic dysfunction and focused on dysuria. This differs from the findings of the main study,^10^ which analyzed all physicians enrolled in the study. Intervention of medications for dysuria may in themselves increase caregiver satisfaction, although their impact on the burden of caregiving was not evaluated in this study. Collectively, it is important for attending physicians to pay attention to patient satisfaction with medications for parkinsonism, in addition to those for autonomic dysfunction and sleep‐related disorders, when considering both treatment needs and satisfaction with medication.

The present study has some limitations. A literature search was conducted before the start of this study to find articles discussing all the clinical manifestations of DLB; however, no such articles were found. Based on a consensus between the study investigators, 52 common symptoms of patients with DLB considered to be clinically important were selected. However, it should be noted that some clinically important symptoms may have been inadvertently omitted from this study. The evaluation of satisfaction with medication in the present study has not been validated. This study is limited by the inherent limitations of a questionnaire survey. The study was conducted in patients with cognitive impairment; thus, the reliability of the patients' answers should be considered with caution. Patients with a sleep‐related disorder were defined as having an NPI‐12 night‐time behaviour sub‐item score ≥1 in this study. Therefore, restless legs syndrome and periodic limb movement disorder were not included as sleep‐related disorders. Our results, particularly in regard to satisfaction with medication for dysuria, should be interpreted with care, considering the small sample size. This study was not a primary objective of the overall study, although it was a pre‐specified subanalysis. Therefore, studies with a larger sample size are needed to assess patients with dysuria. Finally, this study was conducted during the COVID‐19 pandemic, which may have affected the results considering the increased level of anxiety and distress among patients and caregivers during that time.

In conclusion, there was no difference in the satisfaction with medication for symptom domains other than parkinsonism and dysuria among patients with DLB, their caregivers, and their attending physicians. Given that patients' satisfaction with medication for parkinsonism was significantly lower than that of physicians', it is suggested that physicians should pay more attention to patients' satisfaction with medication and prevent undermedication for parkinsonism. Given that caregivers' satisfaction with medication for dysuria was higher than that of physicians, it is also suggested that physicians should pay special attention when they interview caregivers regarding their satisfaction with medication for dysuria, although a larger sample size is necessary to clarify this.

## AUTHOR CONTRIBUTIONS

ST, YM, MH, and MI contributed to the study design, conduct, or collection. All authors contributed to the data analysis and interpretation and to writing or reviewing the manuscript. All authors gave their final approval of the manuscript for submission.

## DISCLOSURE

This study was funded by Sumitomo Pharma Co., Ltd. ST is an employee of Sumitomo Pharma Co., Ltd. YM has received fees for travel expenses from Sumitomo Pharma Co., Ltd.; research funding from Sumitomo Pharma Co., Ltd., and Kamiyama Mfg., Co., Ltd.; and lecture fees from Eisai Co., Ltd., and Mochida Pharmaceutical Co., Ltd. MH has received fees for travel expenses from Sumitomo Pharma Co., Ltd.; and lecture fees from Sumitomo Pharma Co., Ltd., and Eisai Co., Ltd. HY is an employee of 3H Medi Solution Inc. MI has received research funding from Sumitomo Pharma Co., Ltd.; and lecture fees from Sumitomo Pharma Co., Ltd., and Eisai Co., Ltd.

## Data Availability

Data sharing not applicable to this article as no datasets were generated or analysed during the current study.
